# Anxiety among frontline healthcare professionals during the coronavirus pandemic

**DOI:** 10.1192/j.eurpsy.2022.1269

**Published:** 2022-09-01

**Authors:** O. Maatouk, R. Kammoun, I. Kammoun, F. Askri, M. Karoui, H. Nefzi, F. Ellouz

**Affiliations:** 1 Razi Hospital, F Adult Psychiatry Department, Manouba, Tunisia; 2 Razi hospital, Psychiatry G, manouba, Tunisia; 3 Razi Hospital, Avicenne Psychiatry Department, Mannouba, Tunisia; 4 Razi hospital, Psychiatry G Department, Mannouba, Tunisia; 5 Razi hospital, Psychiatry G, denden, Tunisia

**Keywords:** Coronavirus-2019, frontline staff, Anxiety

## Abstract

**Introduction:**

Anxiety has become a topical issue since the arrival of the coronavirus pandemic, especially for frontline healthcare professionals as they deal with patients affected by the Covid-19.

**Objectives:**

Objectify anxiety in frontline medical and paramedical staff and study its associated factors.

**Methods:**

We conducted a national descriptive and analytical cross-sectional study via a survey over a 2-month period from September to October 2020. We used “Beck Anxiety Inventory” to screen anxiety as well as “Brief Cope Scale” to detect probable correlations between anxiety and coping mechanisms.

**Results:**

We collected 78 persons. The mean age was 29.86 years. 35.9% moved out of home. 39.7% worked in Covid units. 7.7% had personal psychiatric history. 76.9% provided direct care to patients with Coronavirus. The frontline staff reported that only 29.5% of patients were stables. Only 48.4% received adequate training of protection against Covid-19. 64.1% of professionals did PCR test and only 16.7% of them tested positive. We objectified an increase of 6.4% in the anxiolytics use. Stigma affected 57.7% of professionals. We highlighted a link between anxiety and social support strategy (p=0.048). 92.3 % of the staff suffered from anxiety according to Beck Anxiety Inventory.

**Conclusions:**

Screening anxiety among frontline medical and paramedical staff might enhance their productivty and thus provide patients with the best care.

**Disclosure:**

No significant relationships.

